# Ethical issues in cluster randomized trials conducted in low- and middle-income countries: an analysis of two case studies

**DOI:** 10.1186/s13063-020-04269-3

**Published:** 2020-04-16

**Authors:** Augustine T. Choko, Gholamreza Roshandel, Donaldson F. Conserve, Elizabeth L. Corbett, Katherine Fielding, Karla Hemming, Reza Malekzadeh, Charles Weijer

**Affiliations:** 1 Malawi-Liverpool Wellcome Trust Clinical Research Programme, Blantyre, Malawi; 2 Department of Infectious Disease Epidemiology, London School of Hygiene and Tropical Medicine, London, UK; 3 Golestan Research Center of Gastroenterology and Hepatology, Golestan University of Medical Sciences, Gorgan, Iran; 4 Department of Health Promotion, Education and Behaviour, University of South Carolina, Columbia, USA; 5 Department of Clinical Research, London School of Hygiene and Tropical Medicine, London, UK; 6 School of Public Health, University of the Witwatersrand, Johannesburg, South Africa; 7 Institute of Applied Health Research, University of Birmingham, Birmingham, UK; 8 Digestive Disease Research Center, Digestive Diseases Research Institute, Tehran University of Medical Sciences, Tehran, Iran; 9 Rotman Institute of Philosophy, Western University, London, Canada

**Keywords:** Cluster randomized trial, Research ethics, Ethics, Low- and middle-income countries, Informed consent, Equipoise, Post-trial access, Research ethics committee

## Abstract

**Background:**

Cluster randomized trials are common in health research in low- and middle-income countries raising issues that challenge interpretation of standard ethical guidelines. While the Ottawa Statement on the ethical design and conduct of cluster randomized trials provides guidance for researchers and research ethics committees, it does not explicitly focus on low- and middle-income settings.

**Main body:**

In this paper, we use the lens of the Ottawa Statement to analyze two cluster randomized trials conducted in low- and middle-income settings in order to identify gaps or ethical issues requiring further analysis and guidance. The PolyIran trial was a parallel-arm, cluster trial examining the effectiveness of a polypill for prevention of cardiovascular disease in Golestan province, Iran. The PASTAL trial was an adaptive, multistage, parallel-arm, cluster trial evaluating the effect of incentives for human immunodeficiency virus self-testing and follow-up on male partners of pregnant women in Malawi. Through an in-depth case analysis of these two studies we highlight several issues in need of further exploration. First, standards for verbal consent and waivers of consent require methods for operationalization if they are to be employed consistently. Second, the appropriate choice of a control arm remains contentious. Particularly in the case of implementation interventions, locally available care is required as the comparator to address questions of comparative effectiveness. However, locally available care might be lower than standards set out in national guidelines. Third, while the need for access to effective interventions post-trial is widely recognized, it is often not possible to guarantee this upfront. Clarity on what is required of researchers and sponsors is needed. Fourth, there is a pressing need for ethics education and capacity building regarding cluster randomized trials in these settings.

**Conclusion:**

We identify four issues in cluster randomized trials conducted in low- and middle-income countries for which further ethical analysis and guidance is required.

## Introduction

Cluster randomized trials (CRTs) are an increasingly important method used in health research, including research conducted in low- and middle-income countries (LMICs). In CRTs, intact social units, or ‘clusters,’ are randomly allocated to intervention and control conditions, and outcomes are usually collected on individual cluster members [1]. Clusters are diverse and include whole communities, neighborhoods, hospitals, clinics and schools. A random sample of CRTs published between 2000 and 2008 revealed that 15% were conducted in LMICs [2] and a review of specific types of cluster trials reveal that a similar, if not greater, number of more recent cluster trials are conducted in LMICs [3]. While there is a growing literature on the ethics of CRTs, [4–7] few articles deal specifically with ethical issues arising in LMIC settings [8–11]. In this article, we discuss ethical issues in two LMIC case studies, the PolyIran trial (Iran) and the PASTAL trial (Malawi).

Compared to individually randomized designs, CRTs are statistically inefficient and more prone to bias [1, 12]. Consequently, the choice of a cluster randomized design must be justified [13]. Public health, health services and health policy interventions are commonly administered at the level of the community, health system or hospital and, as a result, require a cluster randomized design. In other cases, education and training may be provided to health providers to promote evidence-based care. Evaluating these knowledge translation interventions requires a cluster design, as the patients cared for by each physician constitute a cluster. Individual level interventions, such as patient education, may justify the use of a cluster randomized design to prevent contamination—although only when the risk of contamination is high [14]. Sometimes justifications of administrative efficiency or a reduction in costs are used.

CRTs also raise specific ethical challenges that differ from individually randomized designs and complicate the interpretation of standard research ethics guidelines [4]. These ethical challenges include the following. First, CRTs involve groups rather than individuals. As the moral status of groups is often unclear, this complicates whether permission ought to be sought on behalf of the group, as group interests may conflict with individual interests. Second, in CRTs the units of randomization, intervention and outcome assessment may differ. This can complicate the identification of research participants. Third, clusters may be randomized before cluster members can be approached for informed consent for exposure to the intervention. This can further complicate the consent procedure as the role and permissions that gatekeepers are able to provide is often misunderstood. Fourth, the intervention may be delivered to the cluster as a whole, the individual, or both. As cluster level interventions may be difficult or impossible to avoid, this seems to render refusal of study participation meaningless. Nonetheless, informed consent for data collection in such cases may be required.

The Ottawa Statement on the ethical design and conduct of CRTs provides the first international ethics guidance for CRTs [15]. The Ottawa Statement addresses seven broad ethical issues and sets out 15 recommendations for the design and conduct of CRTs (Table 1). It provides guidance on the justification of the use of a cluster randomized design, the need for research ethics committee review, the identification of research participants, obtaining informed consent, seeking gatekeeper permission, benefit–harm analyses, and protecting vulnerable participants. While the Ottawa Statement is intended to have international applicability, the authors acknowledge that “LMIC perspectives were underrepresented...[and] recommend that subsequent revisions include greater LMIC representation” [15]. In what follows, we use the lens of the Ottawa Statement to analyze two CRTs conducted in LMIC settings in order to identify gaps or ethical issues requiring further analysis and guidance. The identified gaps and ethical issues will inform a forthcoming revision process for the Ottawa Statement. As such, we do not seek to provide solutions to these issues in this paper.

**Table 1:** Ottawa Statement on the ethical design and conduct of cluster randomized trials: summary of recommendations

Number	Ethical issue	Recommendation
1	Justifying the cluster randomized design	Researchers should provide a clear rationale for the use of the cluster randomized design and adopt statistical methods appropriate for this design
2	REC review	Researchers must submit a CRT involving human research participants for approval by an REC before commencing
3	Identifying research participants	Researchers should clearly identify the research participants in CRTs. A research participant can be identified as an individual whose interests may be affected as a result of study interventions or data collection procedures; that is, an individual:
		1) who is the intended recipient of an experimental (or control) intervention; or
		2) who is the direct target of an experimental (or control) manipulation of his/her environment; or
		3) with whom an investigator interacts for the purpose of collecting data about that individual; or
		4) about whom an investigator obtains identifiable private information for the purpose of collecting data about that individual.
		Unless one or more of these criteria is met, an individual is not a research participant
4	Obtaining informed consent	Researchers must obtain informed consent from human research participants in a CRT, unless a waiver of consent is granted by an REC under specific circumstances
5		When participants’ informed consent is required, but recruitment of participants is not possible before randomization of clusters, researchers must seek participants’ consent for trial enrollment as soon as possible after cluster randomization—that is, as soon as the potential participant has been identified, but before the participant has undergone any study interventions or data collection procedures
6		An REC may approve a waiver or alteration of consent requirements when 1) the research is not feasible without a waiver or alteration of consent and 2) the study interventions and data collection procedures pose no more than minimal risk
7		Researchers must obtain informed consent from professionals or other service providers who are research participants unless conditions for a waiver or alteration of consent are met
8	Gatekeepers	Gatekeepers should not provide proxy consent on behalf of individuals in their cluster
9		When a CRT may substantially affect cluster or organizational interests, and a gatekeeper possesses the legitimate authority to make decisions on its behalf, the researcher should obtain the gatekeeper’s permission to enroll the cluster or organization in the trial. Such permission does not replace the need for the informed consent of research participants
10		When CRT interventions may substantially affect cluster interests, researchers should seek to protect cluster interests through cluster consultation to inform study design, conduct and reporting. Where relevant, gatekeepers can often facilitate such a consultation
11	Assessing benefits and harms	The researcher must ensure that the study intervention is adequately justified. The benefits and harms of the study intervention must be consistent with competent practice in the field of study relevant to the CRT
12		Researchers must adequately justify the choice of the control condition. When the control arm is usual practice or no treatment, individuals in the control arm must not be deprived of effective care or programs to which they would have access were there no trial
13		Researchers must ensure that data collection procedures are adequately justified. The risks of data collection procedures must 1) be minimized consistent with sound design and 2) stand in reasonable relation to the knowledge to be gained
14	Protecting vulnerable participants	Clusters may contain some vulnerable participants. In these circumstances, researchers and RECs must consider whether additional protections are needed
15		When individual informed consent is required, and there are individuals who may be less able to choose participation freely because of their position in a cluster or organizational hierarchy, RECs should pay special attention to recruitment, privacy and consent procedures for those participants

*CRT* cluster randomized trial, *REC* research ethics committee

## Case study 1

### Summary of the PolyIran trial

Deaths from coronary artery disease are anticipated to increase twofold from 1990 to 2020, and a large majority of the increase is expected to occur in LMICs [16, 17]. Thus, strategies to prevent coronary artery disease that are suitable for implementation in LMICs are a health priority. The polypill concept—a fixed-dose combination pill of established generic drugs—may simplify and provide a more acceptable treatment regimen, while preventing substantial numbers of heart attacks and strokes [18]. Furthermore, the availability of most of the polypill components in a generic form may help to reduce its cost, which is especially important in lower-resource settings [19].

The PolyIran trial was designed to assess the effectiveness and safety of the polypill tablet for primary and secondary prevention of cardiovascular disease (Table 2) [20, 21]. The polypill tablet comprises four generic drugs to reduce blood clotting, lower lipids and reduce blood pressure (aspirin, atorvastatin, hydrochlorothiazide, and either enalapril or valsartan). The PolyIran trial is nested within the infrastructure of the Golestan cohort study and will determine the value of the polypill in a real-life setting. The Golestan cohort study was launched in January 2004 to investigate the epidemiology of esophageal cancer in participants 40–75 years old in Golestan province, Iran [22]. Cohort participants who lived in rural areas and were at least 50 years old were eligible for the PolyIran trial. Villages (clusters) were randomly allocated to one of two arms: the polypill arm or the augmented care arm. In each cluster, residents over the age of 50 years were invited to receive a daily polypill tablet and nonpharmacological preventive interventions (polypill arm) or nonpharmacological preventive interventions alone (augmented care arm). In total, 8410 participants were enrolled from 236 clusters, including 4233 participants (120 clusters) in the polypill arm and 4177 participants (116 clusters) in the augmented care arm. An additional comparison will be made between the two trial arms and Golestan cohort study members who were eligible for, but not selected to participate in, the PolyIran trial but who live in villages not included in the trial (comparator cohort) [20].

**Table 2:** Summary of the key characteristics of the PolyIran trial and PASTAL trial

	PolyIran trial	PASTAL trial
Setting	Golestan province, Iran	Malawi
Design	Parallel-arm, individual-cluster trial	Adaptive, parallel, multiarm, two-stage, individual-cluster trial
Design justification	Avoid contamination	Administrative and logistical reasons
Number of clusters	236 villages	Stage 1: 36 antenatal clinic care days
		Stage 2: 35 antenatal clinic care days
Number of participants	8410	Stage 1: 1007
		Stage 2: 1236
Method of random allocation	1:1 ratio	1:1:1:1:1:1 ratio in stage 1
Data collection procedures	Data collected as part of Golestan cohort study	Review of health records, interviews
Experimental intervention	Polypill plus nonpharmacological prevention	Two oral HIV self-test kits only, or two oral HIV self-test kits with an offer of $3, $10 or lottery incentive conditional on clinic attendance, or followed by a phone call reminder
Control intervention	Nonpharmacological prevention alone	Invitation letter to the male partner offering HIV testing
Primary outcome	Major cardiovascular events	Proportion of male partners who underwent testing for HIV, regardless of serostatus, and linked to clinic for HIV treatment or prevention within 28 days
REC review	Tehran University of Medical Sciences Research Ethics Committee	London School of Hygiene & Tropical Medicine Ethics Committee and College of Medicine Research Ethics Committee
Gatekeepers	Local health care workers (Behvarz), local religious leaders	Local district health officer, health clinic in-charge
Consent model	Verbal informed consent with documentation	Written informed consent from women; waiver of consent for male partners

*REC* research ethics committee

The primary outcome in the PolyIran trial is the occurrence of major cardiovascular events within 5 years of enrolment [20]. Outcomes will be ascertained through the Golestan cohort study that involves collection of these data for all cohort participants through annual telephone contact, home visits, interview and medical record review. Personnel who collected outcome data were blinded to trial participation and treatment arm allocation.

### Ethical issues raised by the PolyIran trial

#### Justification for the use of a cluster randomized design

As cluster randomized designs are statistically inefficient and prone to bias, their use requires justification (Table 1). In this case, residents of the villages in northern Iran have close familial relationships and residents commonly share medicines with one another. Therefore, the issue of contamination was a major concern. A cluster randomized design in which the village was the cluster was chosen to mitigate the risk of contamination through pill sharing [20].

#### Obtaining informed consent from trial participants

Although the PolyIran trial is a CRT, the trial interventions (polypill tablet and nonpharmacological prevention) were offered to all village residents over the age of 50 years. As the study intervention is delivered at the level of the individual, the trial is an individual-cluster trial. In individual-cluster trials, it is typically feasible to obtain the informed consent of participants. Thus, in the PolyIran trial, informed consent was obtained for the polypill and augmented care interventions and use of outcome data.

The high illiteracy rate in the study population (almost 80%) [22] presented a challenge to obtaining informed consent. Research staff who are native Turkmen speakers were trained to obtain informed consent, provided information verbally to participants, and answered any questions. Research staff then documented the disclosure and the participant’s consent (or refusal) to participation in the PolyIran trial.

Members of the Golestan cohort study provided informed consent for the collection and use of data within the cohort study, but they did not provide consent for their data to be used in the PolyIran trial. Consequently, Golestan study cohort members who were randomly selected for the comparator cohort neither provided their permission nor were they aware that their data were to be used in the PolyIran trial. While the outcome data collection was identical between the two studies, both studies have differing purposes and objectives. After deliberation, the research ethics committee responsible for the PolyIran study concluded that no additional consent was required from Golestan study cohort members to use their outcome data in the PolyIran trial.

#### Dealing with current clinical practice in the study population

As with many trials conducted in LMICs, recruitment for the PolyIran trial was not undertaken in conjunction with the participants’ primary or secondary care physician, but rather through a separate and study-specific group of physicians. Some participants were already receiving treatment for cardiovascular disease. For control arm participants, their medications were recorded. However, for intervention arm participants, the researchers sent a letter to patients’ physicians explaining the trial and the composition of the polypill tablet. In case of medication duplication, physician were asked to adjust participants’ prescribed medications accordingly. All changes to medications were recorded for future follow-up and analysis.

#### Post-trial access to the study interventions

It is widely acknowledged that, particularly in an LMIC setting, participants should have access to a trial intervention when it is found to be effective [23]. In the PolyIran trial, if the polypill is shown to be safe and effective, it is anticipated that it will be provided to participants in the polypill and augmented care trial arms for 5 years. However, for how long should the polypill tablet be provided to participants at the expense of the researchers and sponsor? Naturally, there are concerns regarding feasibility of this decision for long-term access.

Added complications follow when considering the issue of whether members of the Golestan cohort study selected for the comparator cohort should be provided with the study intervention should it be found to be effective. Not only does this have larger resource and cost implications, but also as their informed consent for data use in the PolyIran trial was not obtained, comparator cohort participants were not aware of the trial. It was decided that members of the comparator cohort would not be provided with the polypill tablet after completion of the trial.

#### Research ethics committee review

Consistent with other international ethics documents, the Ottawa Statement requires that all CRTs be submitted to a research ethics committee (Table 1). The PolyIran trial was reviewed and approved by the research ethics committee of the Digestive Diseases Research Institute at the Tehran University of Medical Sciences in Iran. In its review, the research ethics committee did not refer to the Ottawa Statement. However, the research ethics committee had reviewed CRTs before and is knowledgeable about cluster randomized designs.

## Case study 2

### Summary of the PASTAL trial

Each year, 1.1 million people die from human immunodeficiency virus (HIV) infection worldwide and 1.9 million people become infected, the majority of whom reside in LMICs [24]. However, little over half of people living with HIV are aware of their HIV status, while many of those who know their status do not start antiretroviral therapy [24]. The benefits of timely initiation of antiretroviral therapy [25] and effective HIV prevention, including voluntary medical male circumcision, [26–29] have changed the emphasis of HIV testing services from learning one’s status to appropriate linkage to care or prevention and retention [24]. However, uptake of HIV testing services and linkage into care or prevention remains below current targets in most LMICs [30, 31]. Major barriers to HIV testing include lack of confidentiality, costs incurred by users, and lack of perceived benefits from accessing HIV testing services [32–36]. The use of financial incentives has been shown to increase the uptake of HIV testing [37, 38] but with mixed results for linkage to post-test services in different settings [39–41].

Building on the successes of HIV self-testing in other populations, [42, 43] the Partner-provided Self-Testing and Linkage (PASTAL) trial was designed to investigate the effect of interventions to increase the uptake of HIV testing and linkage to care or prevention among male partners of pregnant women. PASTAL was a parallel, multiarm, multistage CRT (Table 2). Initial randomization was to six trial arms. The usual care arm involved an invitation letter to the male partner offering HIV testing sent via a pregnant partner accessing antenatal care for the first time. Five intervention arms provided: 1) a self-test kit only; 2) a self-test kit plus a $3 incentive; 3) a self-test kit plus a $10 incentive; 4) a self-test kit plus a lottery to receive $3; and 5) a self-test kit plus a phone call reminder. The cluster was the antenatal care clinic day (e.g., Monday, Tuesday), chosen on the basis of how antenatal care services are structured.

The trial randomized 36 clusters in the first stage in which 93% (1007/1084) of eligible women were recruited between August and November 2016. An independent set of 35 clusters were randomized for the second stage with 97% (1236/1275) of eligible women recruited between January and May 2017. The two-stage design allowed a predefined interim analysis followed by predefined changes to the trial design including sample size recalculation and dropping poor-performing trial arms. The primary outcome was the proportion of male partners who underwent testing for HIV (regardless of serostatus) and who were followed up in a clinic for HIV treatment or prevention within 28 days. Male partner clinic attendance meant achievement of the primary outcome, with follow-up in-person interview with women used to measure secondary outcomes including adverse events.

### Ethical issues raised by the PASTAL trial

#### Justification of the use of an adaptive cluster randomized design

The study interventions (letters, self-test kits and financial incentives) are administered at the level of the individual and thus, in principle, an individually randomized trial is possible. However, for administrative and logistical reasons, and concerns over contamination, a cluster randomized design was preferred [44].

#### Obtaining informed consent from trial participants

Although the PASTAL trial is a CRT, the unit of intervention is the individual. Accordingly, the written informed consent of all female participants was obtained. On each day of recruitment, trial staff provided women attending the antenatal care clinic with general information about the trial. The intervention to which the antenatal care clinic day had been randomized was not disclosed to the woman. After women completed their antenatal care visit, they met one-on-one with a trial staff member who screened them for eligibility and completed the informed consent process and disclosed the trial arm.

While male partners of the female participants were the direct targets of the intervention, their consent to participate was not obtained. A waiver of consent for male participants was sought and granted by the research ethics committee. The waiver of consent was sought for logistical reasons (male partners typically did not accompany women to the antenatal clinic) and scientific reasons (willingness to consent was thought to be correlated with subsequent clinic attendance, the primary outcome measure). A number of concerns might be raised about this approach. First, it compromises the autonomy of male partners. Second, it may pose a risk to women in the study if male partners discover they were enrolled without their consent, as the prevalence of intimate partner violence is high in the region [45].

#### Having conducted an interim analysis that showed the usual care arm to be suboptimal, was there equipoise to justify continuation to the second stage of the trial?

The original sample size calculation assumed that the usual care arm would have 25% of male partners achieving testing for HIV [46]. The analysis at the end of stage one revealed this proportion in the usual care arm was just 13%. Given that so few men in the usual care arm were undergoing HIV testing, data safety and monitoring board members debated whether the usual care should be carried forward to the second stage or dropped. The need to include a usual care arm is a contentious issue not only in adaptive trial designs; not including a usual care arm would mean the trial would be unable to provide a direct comparison to the care provided routinely outside of the trial, limiting its applicability to the local population.

#### Post-trial access to study interventions

In the past 10 years, there has been considerable interest in behavioral economics interventions, such as financial incentives, to improve HIV outcomes [47]. The PASTAL trial found that only the two fixed financial incentive interventions of $3 and $10 significantly improved the primary outcome. However, local policy makers remain unwilling to scale up these programs despite the strong evidence in favor of their effectiveness. This raises the question of sustainability or indeed whether there is social value in conducting trials with these types of interventions in contexts where routine implementation cannot be guaranteed. To what degree a trial must begin with assurances that effective interventions will be implemented is a question requiring further reflection and guidance.

#### Research ethics committee review

The PASTAL trial was reviewed and approved by the College of Medicine Research Ethics Committee in Malawi, and the London School of Hygiene & Tropical Medicine Ethics Committee in London, UK. In their reviews, neither research ethics committee referred to the Ottawa Statement. However, both research ethics committees have reviewed CRTs previously and are knowledgeable about cluster randomized designs.

## Discussion

The Ottawa Statement on the ethical design and conduct of CRTs provides ethical guidance for CRTs but does not explicitly consider issues specific to the LMIC setting. Our ethical analysis of two case studies reveals gaps in the Ottawa Statement and shows that further work is required on ethical issues of CRTs in the LMIC setting. Here we briefly highlight four issues. First, obtaining informed consent remains a challenge in some LMIC settings. The ethical principle of respect for persons supports a general requirement that researchers acquire the informed consent of research participants. In some LMICs, however, low levels of literacy may impede the ability of researchers to seek and obtain informed consent in writing [48]. In other settings, the fact that potential participants fail to distinguish research activities from clinical care or that they possess different health concepts poses a barrier to valid informed consent [49].

In the PolyIran trial, high rates of illiteracy necessitated the use of verbal consent obtained and documented by local research staff. However, issues such as when is verbal consent acceptable and what practices ought to be in place to ensure accurate communication of study information and voluntary consent to study participation remain unanswered. In the PASTAL trial, pregnant women provided written informed consent to study participation, but their male partners did not. Criteria for a waiver of consent (socially valuable research, minimal risk, and infeasibility with consent) require considerable interpretation. How ought minimal risk be understood (e.g., is the possibility of partner violence consistent with minimal risk), and when is a study ‘infeasible’ if consent is required (e.g., lower recruitment, additional research staff required, cost); all these require due consideration if they are to be implemented reasonably and consistently.

Second, the choice of an ethically appropriate control arm remains contentious in LMIC settings. The ethical principle of beneficence requires that the potential benefits and risks of participation stand in reasonable relation. This means, in part, that the study intervention and control need to be consistent with equipoise. The short-course zidovudine trial to prevent maternal–fetal transmission of HIV in sub-Saharan Africa raised prominently the issue of appropriate level of care for control groups in trials conducted in LMICs [50]. Should those in the control arm receive augmented care, care as defined by national (or even international) standards, or locally available care [51]?

The PolyIran trial employed both an augmented care arm with nonpharmacological prevention for cardiovascular disease and a nonrandomized comparator cohort who received no study interventions. The PASTAL trial employed a usual care arm but discovered at a planned interim analysis that local care was producing very poor results in terms of follow-up testing of male partners (13% compared to an expected 25%). The issue of locally available care versus care mandated by national or international standards is an especially pressing issue in CRTs of implementation interventions. In order to obtain evidence that an intervention will improve care at the local level, locally available care is needed as the comparator group.

Third, post-trial access is an important issue for CRTs in LMIC settings. The ethical principle of justice requires that the burdens and benefits of study participation ought to be distributed equitably. Post-trial access to study interventions that are proven effective is particularly important in LMIC settings [52]. The Ottawa Statement says that research ethics committees “should consider whether and when the control clusters will receive the study intervention if the study intervention is shown to be effective” [15]. More recent guidance from the Council for International Organizations of Medical Sciences requires researchers and sponsors to “make every effort, in cooperation with government and other relevant stakeholders, to make available as soon as possible any intervention or product developed, and knowledge generated, for the population or community in which the research is carried out” [23].

However, further guidance is required. The PolyIran trial raises the issue of how broadly post-trial access should be conceived—to trial participants only or all those who contributed data to the analysis? The PASTAL trial highlights the difficulty of ensuring access to effective interventions after the trial.

Fourth, there is a pressing need for ethics education and capacity building regarding CRTs in LMIC settings. The need for capacity building and education in research ethics in LMICs has been recognized for some years. Millum and colleagues observe that:

Global health research will not thrive without a knowledgeable global discussion of the ethics of the research to shape its course. Given the expected further increases in the health research hosted by LMICs, more capacity will have to be built in order to ensure that human subjects protections are maintained even at present levels… Neglected disease trials in disease-endemic countries pose particularly difficult ethical challenges [53].

Innovative or unfamiliar trial designs also pose ethical challenges for researchers and research ethics committees in LMICs. CRTs are increasingly used in LMICs and they raise issues that complicate the interpretation of standard ethics guidelines.

While the Ottawa Statement on the ethical design and conduct of CRTs provides useful guidance, [15] the research ethics committees reviewing the PolyIran and PASTAL trials did not refer to the document. In our experience, the Ottawa Statement is not widely known or used within LMICs. Thus, education on ethics issues in CRTs and available guidance is a priority for health research in LMICs. Web-based educational materials on the ethics of CRTs with LMIC examples would provide a widely accessible resource. Regional workshops for researchers and research ethics committees would provide the opportunity for deeper discussion of issues and learning. Finally, training fellowships would allow researchers and ethics committee members the opportunity to develop specific projects with internationally recognized experts.

## Conclusion

Our ethical analysis of the PolyIran and PASTAL trials reveals gaps in the Ottawa Statement and shows that further work is required on ethical issues of CRTs in the LMIC setting.

First, standards for verbal consent and waivers of consent require methods for operationalization if they are to be employed consistently. Second, the appropriate choice of a control arm remains contentious, and the acceptability of locally available care as the comparator in implementation CRTs, especially when such care falls below national standards, should be assessed. Third, as it is often not possible to guarantee post-trial access to study interventions, clarity on stakeholder obligations is needed. Fourth, there is a pressing need for ethics education and capacity building on CRTs. These identified gaps and ethical issues will inform a forthcoming revision process for the Ottawa Statement.

## Abbreviations

CRT: Cluster randomized trial
HIV: Human immunodeficiency virus
LMIC: Low- and middle-income country
